# Efficient slice anomaly detection network for 3D brain MRI Volume

**DOI:** 10.1371/journal.pdig.0000874

**Published:** 2025-06-20

**Authors:** Zeduo Zhang, Yalda Mohsenzadeh

**Affiliations:** 1 Department of Computer Science, Western University, London, Ontario, Canada; 2 Vector Institute for Artificial Intelligence, Toronto, Ontario, Canada; University of Illinois Urbana-Champaign, UNITED STATES OF AMERICA.

## Abstract

Current anomaly detection methods excel with benchmark industrial data but struggle with natural images and medical data due to varying definitions of ‘normal’ and ‘abnormal.’ This makes accurate identification of deviations in these fields particularly challenging. Especially for 3D brain MRI data, all the state-of-the-art models are reconstruction-based with 3D convolutional neural networks which are memory-intensive, time-consuming and producing noisy outputs that require further post-processing. We propose a framework called Simple Slice-based Network (SimpleSliceNet), which utilizes a model pre-trained on ImageNet and fine-tuned on a separate MRI dataset as a 2D slice feature extractor to reduce computational cost. We aggregate the extracted features to perform anomaly detection tasks on 3D brain MRI volumes. Our model integrates a conditional normalizing flow to calculate log likelihood of features and employs the contrastive loss to enhance anomaly detection accuracy. The results indicate improved performance, showcasing our model’s remarkable adaptability and effectiveness when addressing the challenges exists in brain MRI data. In addition, for the large-scale 3D brain volumes, our model SimpleSliceNet outperforms the state-of-the-art 2D and 3D models in terms of accuracy, memory usage and time consumption. Code is available at: https://github.com/Jarvisarmy/SimpleSliceNet.

## Author summary

In this work, we address the need for more effective methods to identify unusual patterns in brain MRI scans, which are crucial for diagnosing neurological conditions. Traditional techniques often struggle with the high diversity in normal brain appearances and are usually slow and demanding on computing resources. To overcome these challenges, we developed SimpleSliceNet, a streamlined approach that simplifies the analysis without sacrificing accuracy. Our method operates more quickly and requires fewer computational resources than older methods, making it highly practical for clinical settings. By improving the detection of anomalies in brain images, our method holds promise for earlier and more accurate diagnoses of neurological issues, which can directly enhance patient care. Additionally, our approach reduces the technical barriers often faced in medical imaging, potentially broadening the access to high-quality diagnostic techniques across different healthcare environments. Our findings demonstrate that our approach surpasses current leading methods, suggesting a significant step forward in the field of medical imaging.

## Introduction

Anomaly detection (AD) aims to identify defects or outliers within datasets, commonly applied across natural, industrial, and medical data. Unsupervised anomaly detection (UAD) [1–4] has gained attraction due to the diverse and unpredictable nature of anomalies. In addition, the scarcity of anomaly samples poses challenges for model adaptation, often leading to overfitting. Thus, UAD, which requires no anomaly sample training, is often preferred.

Magnetic Resonance Imaging (MRI) of the brain is a non-invasive medical imaging technique used to produce detailed images of brain structure and function. Anomalies in brain MRI indicate deviations from typical or healthy brain structure or function, including abnormalities like tumors, cysts, or vascular malformations. While the MVTecAD production line dataset [5] is commonly used to evaluate state-of-the-art (SOTA) anomaly detection models for images, it differs from brain MRI data. In the MVTecAD dataset, normal objects exhibit consistent patterns characterized by concentrated normal features, and any deviations from these patterns are identified as anomalies. However, brain MRI structure can vary due to differences among patients, biological changes, technical factors, patient movement, and calibration and correction processes.

While the SOTA methods [2,6–9] have achieved nearly perfect outcomes on industrial data (e.g. MVTecAD), they still face challenges when applied on natural images and medical data. Prominent models, including reconstruction-based models [10], embedding-based models [2,6,7], and student-teacher models [1,11,12], often employ feature extractors pre-trained on ImageNet. This dataset which includes natural object images features a domain distinct from medical data. Furthermore, models specifically designed for 3D data are scarce. The most common method to perform anomaly detection on 3D brain MRI volumes are reconstruction-based frameworks with 3D Convolutional Neural Network (3D-CNN) [13,14]. These models require lots of memory to store the 3D kernels, consume time to converge, and typically produces noisy results, necessitating additional post-processing steps. Few supervised anomaly detection methods [15,16] for 3D data have also been proposed, presenting the challenge of costly annotation for such data. Undoubtedly, they also encounter the same issue of memory and time.

To harness the capabilities of existing 2D anomaly detection architectures, we found that methods using 2D-slice models are more practical [17,18]. These models process each slice in the volume sequentially and aggregate the information afterward. Considering the considerable volume of 3D data, certain complex and computationally demanding models may not be feasible. We seek a simple yet effective model suitable for both 2D and 3D brain MRI data due to the significant volume of 3D data and computational constraints. Recently presented SimpleNet [6] emerges as a promising candidate, being lightweight, efficient, and not requiring anomalies for training. However, its instability in certain scenarios and applicability only to single-structure images, like those in industry production lines, pose challenges. To adapt SimpleNet to the diverse features of brain MRI, particularly in distinguishing between normal and abnormal features, modifications are necessary.

The primary objective of this study is to find an efficient model for detecting anomalies in brain MRI images and apply it to analyze 3D brain MRI volumes. We propose a novel anomaly detection model for medical data inspired by SimpleNet framework [6] and evaluate the effectiveness and performance of our model on both 2D and 3D brain MRI datasets for this purpose. To ensure adaptability to medical data, we fine-tuned the feature extractor (which was pre-trained on ImageNet) on an independent medical dataset with an unsupervised Encoder-Decoder Contrast (EDC) [10] technique. This step also reduces computational cost during training of our SimpleSliceNet model. Additionally, we propose that the discriminator in SimpleNet [6], which utilizes truncated L1 loss, functions as a complete pushing mechanism that could potentially contribute to a higher false positive rate. To tackle this issue, we instead introduce a Semi-Push-Pull contrastive Loss to our model, which selectively modifies ambiguous regions to establish an ambiguous boundary between normal and abnormal features, thereby reducing false positives. While anomalies in SimpleNet are synthesized by adding noise to the feature, categorizing those with little variation solely as anomalies may lead to a high false positive rate. We discover that the Semi-Push-Pull Mechanism proves beneficial in addressing this issue by softly modifying the normal region with a margin. So that the anomalous features won’t be forced to deviate from the normal distribution too much. Instead, an ambiguous boundary between the normal and abnormal features is established. And so those synthesized anomalies that are close to anomalies still have high likelihood. Furthermore, we found that the training process in SimpleNet is unstable which might be caused by the unclear balance between projection and discriminator layers and the full pushing mechanism. We show that a single Normalizing Flow (NF) can address this issue and outperform without additional projection layers. Finally, we demonstrated the effectiveness of our model in detecting anomalies in 3D volumes using a slice-by-slice approach, leveraging the idea of 2D-Slice models. Few papers focus on applying embedding-based methods to medical datasets due to the aforementioned challenges and issues presented in Discussion, especially for 3D brain MRI volumes, where conventional 2D models cannot be directly applied. The contributions of this paper are summarized as below:

1. We design new lightweight embedding-based model with a pre-trained features extractor and an additional semi-push-pull contrastive loss to detect and localize the anomalies on brain MRI dataset and outperform the (SOTA) models.
2. We highlight the effectiveness of fine-tuning the feature extractor model using a separate MRI dataset, a choice made to ensure the pre-trained model remains uninformed about knowledge of the training data. This approach significantly enhances feature projection to the target space compared to alternative methods. Moreover, it offers notable advantages in terms of memory and time efficiency, particularly crucial for handling extensive 3D volumes.
3. We conducted extensive experiments comparing our model with SOTA 2D and 3D methods, demonstrating that our model achieves SOTA performance in terms of accuracy, memory usage and time consumption. Additionally, we evaluated the performance by individually removing each component from our model, quantitatively demonstrating the significance of each component.

## Material and methods

### Problem setup

In this study, we focus on unsupervised brain MRI anomaly detection and localization. Our model processes 3D MRI volumes and performs anomaly localization by assigning an anomaly score to each voxel. Based on this pixel-level anomaly map, a slice is classified as anomalous if it contains at least one pixel with a high anomaly score. This localization-first approach ensures that detection naturally follows from the localization process.

Although our model operates on 3D volumes, we perform evaluation at the slice level to ensure fair comparison with existing 2D datasets and models. We specifically use axial slices, as they are the most common orientation in 2D brain MRI datasets and clinical diagnostics, making our evaluation setup more consistent with real-world applications.

For unsupervised training, we use IXI, a dataset of healthy brain MRI scans. The model is then evaluated on BraTS2021, which provides pixel-level tumor annotations. Since obtaining labeled abnormal MRI data is costly and challenging, this setup enables us to assess the model’s ability to generalize without relying on annotated abnormal samples.

### Dataset

We utilize three publicly available brain MRI datasets: Br35H [19], IXI [20], and BraTS2021 [21–23], each serving a specific purpose. We use the Br35H dataset for fine-tuning the pretrained feature extractor without additional preprocessing, to preserve its original characteristics and ensure that the model does not have prior knowledge of the training and testing data. This approach allows us to adapt the model to MRI data in a generalizable way.

In contrast, the IXI and BraTS2021 datasets are preprocessed with the same steps (e.g., skull-stripping and resampling) to maintain consistency between training and testing data. These preprocessing steps are commonly used in brain MRI studies [13,24]. Skull-stripped helps focus on brain regions, while co-registration standardizes spatial alignment, addressing distribution shifts caused by variations in imaging sources.

**Fig 1:**
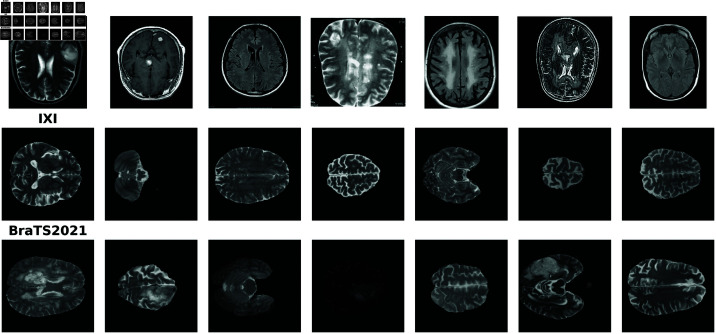
Samples from datasets. This figure displays samples from each dataset used in this study. Each row represents samples from a different dataset, providing a visual comparison of their characteristics and preprocessing differences. Samples from the IXI and BraTS2021 shown in this figure have undergone preprocessing.

- **Br35H** is a 2D brain MRI dataset on Kaggle [19]. It contains 1,500 2-D image slices that are tumorous and 1,500 images that are non-tumorous. We resized the non-tumorous images to 256×256 and used them to fine tune the pre-trained model. The dataset includes T1-weighted and T2-weighted modalities. From Fig 1, we observe that images from Br35H are inconsistent, not skull-stripped, and represent only the central area. Detailed acquisition parameters, such as field strength, repetition time, echo time and voxel resolution, are not explicitly provided in the dataset documentation.
- The **IXI** dataset [20] contains healthy subjects with T1, T2, PD, MRA and DTI modalities. The spatial resolution of all slices is $0.94\;\times \;0.94\;\times \;1.25\;{\text{mm}}^{3}$, with an in-plane matrix size fixed at 256×256, and the number of slice ranged from 28 to 136. We use the T1- and T2-weighted volumes from these 581 and 576 healthy subjects for training. The data were collected from hospitals in London: Hammersmith Hospital using a Philips 3T system, Guy’s Hospital using a Philips 1.5T system and Institute of Psychiatry using a GE 1.5T system. The T2 acquisition parameters for the Philips 3T system are: repetition time = 5725.79 ms, echo time = 100.0 ms, phase encoding steps = 187, echo train length = 16, reconstruction diameter = 240.0 mm, acquisition matrix = 192×187, and flip angle = 90∘. For the Philips 1.5T system, the parameters are: repetition time = 8178.34 ms, echo time = 100.0 ms, phase encoding steps = 187, echo train length = 16, reconstruction diameter = 240.0 mm, and flip angle = 90∘. The parameters for the GE 1.5T system are not available.
- The **BraTS2021** dataset [21–23] comprises a total of 1,251 subjects with glioblastoma, collected from 19 institutions. All volumes are interpolated to a $1\;\times \;1\;\times \;1\;{\text{mm}}^{3}$ resolution, skull-stripped, and cropped to a fixed size of 240×240×155. We use the T1 and T2-weighted volumes for validation and testing. Due to variations in institutions and scanners, acquisition parameters are not publicly available. Within this dataset, the first 251 volumes are used for validation, while the remaining 1,000 are used for testing.

The IXI and BraTS2021 datasets share only T1- and T2-weighted modalities. However, T1 is not suitable for glioblastoma localization due to its low contrast in highlighting abnormal regions, as shown by both quantitative and qualitative results at the pixel level. Since our primary task is anomaly localization, the main experiments and analyses are conducted on T2-weighted volumes. The efficiency study is also based on T2-weighted volumes, as the choice of modality does not affect the results. However, we also provide quantitative and qualitative results for T1-weighted volumes in figures in S1 Fig, S2 Fig and S3 Fig. Unless otherwise specified, all experiments in this paper are conducted on T2 volumes. Samples from these three datasets are shown in Fig 1

### Pre-processing

We followed the 3D brain MRI volume pre-processing procedures in [13], used HD-BET tool [25] to skull-stripped the T2 volumes from IXI, and resample all the 3D volumes from IXI and BraTS2021 to the MNI 1*mm* template using the flirt tool of FMRIB software library [26,27].

### Anomaly scores

For our model, similar to other flow-based models [2,7], the log-likelihood of the distribution can be considered as the normality score, and thus the pixel-level anomaly score is defined as

s(x)=1−exp(logp(x)), (1)

where *s*(*x*) represents the anomaly score for pixel *x*, and logp(x) denotes the log probability of pixel *x*, which is output by the Conditional normalizing flow trained with the loss function in Eq. 11. During training, our model learns to model the normal distribution and assigns high log probabilities to normal samples. During testing, unseen anomalies that deviate from the learned normal distribution are assigned lower log probabilities. Consequently, a feature vector with high anomaly score s(x) indicates a potential anomaly.

The image-level anomaly score is obtained as the maximum pixel anomaly score across the image. Given the extensive volumes in the testing dataset, we evaluate the metrics at the pixel level for each volume. This process involves normalizing the pixel-level anomaly score within each volume, calculating the metrics for each volume, and then computing the mean of these metrics across all volumes to obtain the final results. For image (slice)-level evaluation, we normalize and calculate the metrics across the entire testing dataset. Our approach to image-level evaluation differs slightly from the method presented in [13], which normalizes the normality scores within each volume. A potential issue with normalizing within a single volume is that if a normal volume were present in the testing set, its minimal anomaly score could be artificially inflated, leading to bias. To mitigate this, we choose to perform evaluation across the entire dataset rather than normalizing within each volume. While our testing set does not contain normal volumes, this dataset-level normalization approach provides a more robust and generalizable evaluation. Additionally, there is no concern regarding comparison with other models, as all methods in our study follow the same evaluation protocol.

### Evaluation metrics

In our paper, we utilize a uniform set of metrics to evaluate the performance of our model at both the image level and pixel level. We assess using Area Under the Receiver Operating Characteristic Curve (AUROC), Area Under the Precision-Recall Curve (AUPRC), accuracy (ACC), specificity (Spec), precision (Prec), F1 score (F1), and Maximum Dice Score (⌈DICE⌉). These metrics allow us to comprehensively measure the model’s ability to distinguish between positive and negative instances, balance precision and recall, and achieve high accuracy across different thresholds. To optimize performance evaluation, we dynamically determine the F1 threshold that maximizes F1 score, striking a balance between precision and recall, thus offering an optimal threshold. We use this F1 threshold to achieve the best accuracy, specificity, precision and F1 score.

The AUROC quantifies the model’s capacity to differentiate between classes under varying thresholds, offering a broad view of classification performance. AUPRC is particularly valuable in scenarios with class imbalances, as it focuses on the precision-recall trade-off, providing insights into the model’s effectiveness in identifying true positives amidst a large number of false positives.

Specificity and precision highlight the model’s accuracy in identifying true negatives and true positives, respectively, while the F1 score and Maximum Dice Score offer insights into the harmonic mean of precision and recall, and the spatial overlap accuracy between predicted and actual regions. The Maximum Dice Score allows for the assessment of the model’s optimal performance under the best possible conditions. It is a critical measure in fields like medical imaging, where precise segmentation of anatomical structures is essential for accurate diagnosis and treatment planning.

For the pixel-level evaluation, all the aforementioned metrics apply, with the addition of the per-region-overlay (PRO) metric, which evaluates the spatial correspondence between predicted and ground truth regions, enhancing our understanding of the model’s localization accuracy. Unlike the image-level evaluation, PRO specifically helps in assessing the effectiveness of our model in segmenting and localizing regions at the pixel level.

We observe that the ⌈DICE⌉ score is exactly the same as the maximum F1 score. This similarity arises because both metrics assess the balance between precision and recall in binary classification and segmentation tasks. Therefore, for the results presented in this paper, we use only the ⌈DICE⌉ score.

### Network architecture and training setup

**Fig 2:**
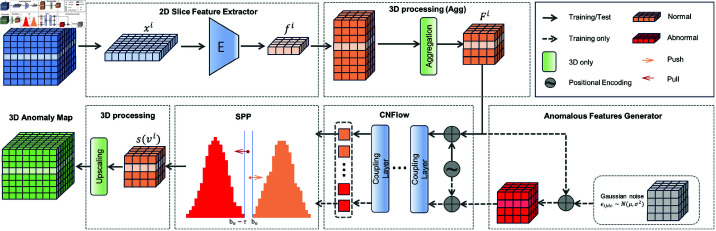
SimpleSliceNet overall architecture. Overview of the proposed SimpleSliceNet. The slice encoder E utilizes a backbone pre-trained on ImageNet to extract multi-layer feature maps $f_{i}$ at low resolution. Anomalies are synthesized by introducing Gaussian noise into the feature space. Subsequently, these features are fed into **CNF** to estimate the log likelihood of the normal and anomalous distribution, and employ a contrastive loss to softly pull the normal features together and push abnormal features away. Our SimpleSliceNet extracts slice features using the slice encoder and aggregates the resulting slice features via permutation invariant operations to achieve the features $F^{i}$ of the volume $v^{i}$ in low resolution. We process the feature vector of each voxel exactly like what we do on 2D slices to obtain anomaly map $s(v^{i})$. We finally upscale the anomaly map to the same resolution as that of the original volume.

Our 2D slice encoder consists of a neural network backbone pre-trained on ImageNet, and conditional NF. The 3D brain MRI volume is processed by extracting slice features using 2D slice encoder and aggregating them to utilize depth-wise information. Fig 2 shows the detail of our model architecture.

For all purpose, we use the WideResnet50 pre-trained on ImageNet [28] as the backbone which is further fine-tuned using EDC. More detail about the backbone model and its fine-tuning is presented in the Encoder-decoder contrast. Through an ablation study in [6] and our own experiments, we determined that utilizing features from 2nd and 3rd intermediate layers yields the best performance. The anomalies are synthesized by adding i.i.d Gaussian noise $𝐍(0,\sigma^{2})$, where σ is set to 0.06 in BraTS experiments. In the condition NF architecture, we employ 8 coupling layers, with each layer consisting of two linear layers and one ReLU activation layer. The target embedding dimension is set to 512. The parameters of the CNF are optimized using an Adam optimizer with a learning rate to 0.001. For fair comparison, the batch size of all 3D models are 1 (volume), and the batch size of all 2D models are 96 slices (one volume).

### Slice feature extractor

We employ a model pre-trained on ImageNet to serve as a feature extractor, extracting multi-layer features which is used in [6,8]. Anomalies are generated by introducing Gaussian noise to these extracted features inspired by [6].

We denote training and testing sets as *I*_*train*_ and *I*_*test*_, respectively. The training dataset (*I*_*train*_) contains only normal samples and the testing dataset (*I*_*test*_) contains a mixture of normal and abnormal samples. Each image from the dataset is defined as $x_{i}\in I_{train}\cup I_{test}$ where $x_{i}\in {\mathbb{R}}^{3\;\times \;H\;\times \;W}$. We passed in the samples into a neural network backbone *B* to extract features. Then we define the output from the intermediate layer l∈L of the backbone as $x^{l,i}\sim B^{l}(x_{i})$, where $x^{l,i}\in {\mathbb{R}}^{C_{l}\;\times \;H_{l}\;\times \;W_{l}}$ and $\left(C_{l},\;H_{l},\;W_{l}\right)$ represents the dimensions of the features map from layer *l*. Let’s define $x_{h,w}^{l,i}$ as the feature representation at location (h,w). Since we try to utilize the local environment information, we aggregate the neighborhood with a patch of size *p* to obtain a local feature vector. The local feature vector of image ${\text{x}}^{\text{i}}$ from layer *l* at location (*h*,*w*) is defined as

$$z_{h,w}^{l,i}=Aggregate(\{x_{h^{\prime },w^{\prime }}^{l,i}|h^{\prime }\in [h-\lfloor p/2\rfloor ,\ldots ,h+\lfloor p/2\rfloor ],\;w^{\prime }\in [w-\lfloor p/2\rfloor ,\ldots ,w+\lfloor p/2\rfloor ]\}).$$ (2)

Then, we upscale all the feature maps with different resolution to same higher resolution and concatenate them together. In our case, we choose the dimension size of the first layer $\left(H_{0},W_{0}\right)$. Then the extracted map of each sample $f^{i}\in {\mathbb{R}}^{H_{0},W_{0},C}$ is defined as

$$f^{i}=concat(upscaling(z^{l,i},(H_{0},W_{0}))|l\in L),$$ (3)

where $C=\sum_{l\in L}C^{l}$. We further aggregate the channel information to decrease the channel size to a desired dimension. For convenience and further definition of 3D formulation, we simplify the feature extraction process as

$$f^{i}=E(x^{i}).$$ (4)

### 2D-slice-model and 3D aggregation

Define a brain MRI volume from a dataset *V* as $v^{i}\in {\mathbb{R}}^{H\;\times \;W\;\times \;D}$, we extract the feature maps of each slice $v_{j}^{i},\;j\in \{1,\ldots ,D\}$ from the volume ${\text{v}}^{\text{i}}$ using the 2D feature extractor E mentioned in Slice feature extractor, so that $f_{j}^{i}=E(v_{j}^{i})$. Then we aggregate the features of all slices to achieve the 3D anomaly representation of a volume. Then we define this process as

$$F^{i}=aggregate(\{E(v_{j}^{i})|j\in [1,\ldots ,K]\}).$$ (5)

### Conditional normalizing flow

Normalizing flow [29] aims to represent the complex distributions with a simple one through a flow of successive invertible and differentiable transformations. The fundamental trick that makes NF work is the change of variables. The critical idea of the change of variables is transforming a random variable X into a tractable variable Z using an invertible and differential flow model z=ϕ(x). The log-likelihood of the random variable can be determined by

$$\log p_{X}(x)=\log p_{Z}(\phi (x))+\log\left|det(\frac{\;d\phi (x)}{\;dx})\right|.$$ (6)

To optimize the log-likelihood, it’s equivalently to approximate the target distribution with a flow-based model with parameters Θ by minimizing the reverse KL distance

$$ℒ(\Theta )={𝔼}_{p_{X}(x;\Theta )}\left[\log p_{X}(x;\Theta )-\log p_{X}(x)\right]\;=-{𝔼}_{p_{X}(x;\Theta )}\left[\log p_{Z}(\phi (x))+\log\left|det(\frac{\;d\phi (x)}{\;dx})\right|\right].$$ (7)

We followed [2,7], assumed the simple tractable distribution obey the multivariate Gaussian distribution and rewritten the Eq (7) as

$$ℒ(\Theta )=-{𝔼}_{p_{X}(x;\Theta )}\left[\frac{d}{2}\log2\pi +\frac{1}{2}\phi (x{)}^{T}\phi (x)-\log\left|det(\frac{\;d\phi (x)}{\;dx})\right|\right],$$ (8)

where *d* denotes the dimensionality of the features. For the transformation, we used coupling layers with fully connected layer which doesn’t contain spatial information. In line with previous works [2,7], we employ conditional NF to integrate 2D positional encoded information.

### Encoder-decoder contrast

When utilizing pre-trained ImageNet models in the medical domain, one of the challenges is the disparity between the distribution of the ImageNet dataset and the target dataset. Common methods to address this issue typically involve fine-tuning the model or training additional layers to project the extracted features into the target space during training process. However, we are exploring an alternative approach to transfer knowledge across domains. We have discovered that fine-tuning the feature extractor before training outperforms other alternatives and can save training time in this case. To keep the standard deviation and discriminative ability of the features from each intermediate layer. We fine-tune the pre-trained model using stop-gradient and global cosine distance proposed by [10]. To maintain the assumption that pre-trained model has no knowledge on the training distribution, we fine-tune the model on an independent 2D MRI images dataset called Br25H [19]. To fine-tune the pre-trained model on target dataset, [10] create a decoder with a reverse architecture of the pre-trained model to reconstruct the images using encoder-decoder framework. For each l-th intermediate layer where l∈L, they denote $f_{E}^{l},f_{D}^{l}\in {\mathbb{R}}^{H_{l},W_{l},C_{l}}$ as the features maps from the l-th layer of the encoder and decoder respectively, where $\left(H_{l},W_{l},C_{l}\right)$ is the dimension of the feature map. Then the reconstruction residuals are minimized by the global cosine distance loss

$${ℒ}_{global}=\sum_{l=1}^{L}1-\frac{sg(F(f_{E}^{l}{)}^{T})\cdot F(f_{D}^{l})}{sg(\|F(f_{E}^{l})\|)\|F(f_{D}^{l})\|},$$ (9)

where *sg* represents the stop-gradient operation, and *F* denote a flatten operation that casts the 2D feature map into feature vector. The stop-gradient operation won’t affect the forward process and will consider the encoder parameters as constant while optimized them through the gradient from the decoder. Authors of [10] proved that the stop-gradient operation can help to find discriminative features and the global cosine distance can address the instability problem. This loss is used to fine-tune the pre-trained feature extractor during the pre-training stage and is excluded from the main anomaly localization training, as the encoder remains frozen during this phase.

### Loss function

Using discriminator to classify the normal features and noisy features (synthesized anomalies) may cause some false positive. Instead, to refine the boundary of normal features, we employ a Triplet Loss [30] to encourage a more compact feature space, softly refining the boundary between normal and anomalous regions. In Study on contrastive losses, we study other contrastive loss including Pairwise Ranking Loss [31] and Boundary-guided Semi-Push-Pull Loss [7].

Given a feature vector *f*_(*h*,*w*)_, we synthesize anomalies by adding Gaussian noise, generating a perturbed feature vector ${\tilde{f}}_{\left(h,w\right)}$. We associate the log probabilities of normal and perturbed features as $\log p_{i}$ and $\log q_{i}$, respectively. To refine the boundary of normal features, we employ a triplet loss defined as

$${ℒ}_{triplet}=\sum_{i}^{N}max(0,||\log p_{i}-\log p_{j}|{|}_{2}^{2}-||\log p_{i}-\log q_{i}|{|}_{2}^{2}+\tau ),$$ (10)

where $||\;\cdot \;|{|}_{2}^{2}$ denotes the squared Euclidean distance, and τ is the margin which is set to 1.0 in our model. The final objective function is to maximize the log-likelihood of the normal samples Eq (8) and to minimize the Triplet loss. The final loss function is

$$ℒoss=ℒ(\Theta )+{ℒ}_{triplet}.$$ (11)

## Results

In this study, we used the healthy subjects from IXI for training and unhealthy brains from BraTS2021 for validation and testing. The pre-processed T2 volumes from both datasets were center-cropped to 192×192×96 and the intensity range was rescaled to [0,1]. The preprocessed T1 volumes from both datasets were resized and padded to 224×224×160 to include the whole brain. Our model was benchmarked against the latest 3D models specifically designed for brain MRI data. Additionally, we explored a straightforward approach of processing brain MRI volumes slice-by-slice using 2D methods, allowing us to also compare our model against SOTA 2D methods.

### Comparison with existing methods

Current SOTA anomaly detection methods can be broadly categorized into reconstruction-based, embedding-based, and synthesis-based approaches. Additionally, memory-bank and knowledge-distillation methods constitute two further subcategories. To ensure a comprehensive comparison, we evaluate our model against a representative 2D SOTA method from each category, as well as a 3D reconstruction-based model, given that other categories remain underexplored in the 3D setting. A detailed discussion of these categories, their underlying principles, and key assumptions is provided in Discussion.

- **EDC** [10] is a reconstruction-based anomaly detection method for medical images utilizing an encoder-decoder architecture. The encoder is initialized with ImageNet-pretrained weights and refined through a global cosine loss and a stop-gradient mechanism to better align with the medical domain. Our approach leverages its global cosine loss to fine-tune the feature extractor for improved adaptation. Compared to EDC, which primarily detects anomalies via reconstruction error, our method directly models the normal feature distribution using NF, allowing for a more flexible representation of normal patterns.
- **DAE** [24] is a reconstruction-based method for brain MRI anomaly detection, employing a denoising U-Net to learn normal patterns and detect anomalies via reconstruction error.
- **AE-FLOW** [32] is a reconstruction-based approach for medical images, incorporating a NF within an encoder-decoder framework to model the latent distribution.
- **3D-AE** [13] is a 3D reconstruction-based method designed for brain MRI, utilizing an encoder-decoder structure.
- **SimpleNet** [6] is an embedding-based method that classifies embeddings of normal features and their noise-perturbed counterparts. The underlying assumption is that adding noise to the normal embedding space generates near-distribution anomalies, aiding in learning the normal feature boundary. Similar to SimpleNet, our method leverages feature perturbation to synthesize anomalies. However, instead of classifying perturbed embeddings, we model the normal feature distribution using NF and refine it with contrastive loss, which provides a probabilistic interpretation of anomaly scores
- **BGAD** [7] is a semi-supervised embedding-based method that models normal distributions using conditional NF and introduces a semi-push-pull loss to explicitly delineate normal and abnormal features. Since our method also leverages NF and examines the effect of the semi-push-pull loss, we include BGAD for comparison. In experiments, ${\text{BGAD}}^{\text{w/o}}$ refers to its unsupervised variant, which excludes labeled anomalies and the BG-SPP loss, while ${\text{BGAD}}^{100}$ denotes the semi-supervised version with 100 known anomalous slices.
- **CFLOW-AD** [2] is another embedding-based method employing conditional NF with multi-scale generative decoders to model normal distributions.
- **CutPaste** [33] is a synthesis-based method that trains a discriminator to classifies normal and synthetically generated anomalous images. The discriminator is then used to classify anomalies during testing.
- **PatchCore** [8] is a memory-bank method that detects anomalies by measuring distances between test samples and a compact set of ImageNet-pretrained normal embeddings.
- **RD++** [12] is a knowledge-distillation method using a teacher-student architecture. The teacher, pretrained on ImageNet, guides the student to mimic its outputs on normal images. Anomalies are identified by discrepancies between student and teacher predictions during inference.

### Localization quantitative results

Table 1 displays the results of pixel-level metrics for all the models. According to the results, our method achieves the best performance, while **PatchCore** and **RD++** show competitive performance in terms of AUROC. However, there is a significant improvement on AUPRC, PRO and ⌈DICE⌉ compared to these methods. As discussed in Evaluation metrics, these three metrics are particularly crucial and representative in tasks involving imbalanced anomaly segmentation. The quantitative results for T1 volumes are also presented in the Table 2 for high-performance models, for further information.

**Table 1:** Quantitative results at pixel level for all models on T2 volumes. This table presents the Quantitative results of all models at the pixel level, providing a comprehensive overview of each model’s effectiveness in anomaly localization. *Ours* denotes our method variant with Triplet Loss.

	AUROC	AUPRC	PRO	⌈ DICE⌉	Spec	ACC	Prec
SimpleNet	86.9	23.31	61.05	28.06	24.27	91.84	93.09
${\text{BGAD}}^{\text{w/o}}$	77.88	7.82	37.81	13.4	8.82	79.09	79.7
${\text{BGAD}}^{\text{100}}$	92.04	35.37	73.13	37.79	35.26	95	96.24
CFLOW-AD	83.21	13.29	46.34	19.87	14.3	82.68	83.44
EDC	88.77	32.86	68.84	37.74	34.6	90.57	91.35
DAE	87.89	16.30	75.08	24.88	19.95	89.40	90.42
AE-FLOW	82.28	12.91	68.85	19.84	14.53	81.53	82.12
CutPaste	73.15	3.64	28.53	7.29	4.5	87.21	88.12
PatchCore	95.51	44.75	76.44	48.52	44.26	96.52	**97.42**
RD++	95.25	38.50	74.99	44.07	37.99	**95.75**	96.64
3D_AE	86.69	18.03	73.42	25.79	20.01	88.37	89.19
Ours	**95.55**	**51.33**	**76.98**	**51.07**	**48.5**	95.67	96.48

**Table 2:** Quantitative results at pixel level for all models on T1 volumes. This table presents the Quantitative results of all models at the pixel level, providing a comprehensive overview of each model’s effectiveness in anomaly localization. *Ours* denotes our method variant with Triplet Loss.

	AUROC	AUPRC	⌈ DICE⌉	Spec	ACC	Prec	
${\text{BGAD}}^{\text{w/o}}$	83.75	5.55	50.28	10.47	6.93	87.06	87.55
EDC	90.66	9.01	73.32	16.3	10.43	87.39	87.56
PatchCore	**95.70**	**22.07**	**83.55**	**30.46**	**24.13**	**95.85**	**96.35**
RD++	93.96	17.49	74.72	28.1	19.96	92.93	93.45
3D_AE	84.58	4.35	64.72	10.29	5.8	83	83.07
Ours	94.70	18.86	78.22	27.84	20.77	94.14	94.53

### Detection quantitative results

Table 3 presents the quantitative results on T2 volumes at the image level for all models. Our methods generally outperforms the other SOTA methods, except for **EDC**, which achieves the best performance across all metrics. However, despite its superior image-level metrics, **EDC** demonstrates poor performance in localization ad lacks stability, as indicated by the numerical results on localization and the qualitative assessments discussed in other subsections. Furthermore, although our model shows visually superior tumor detection compared to **EDC**, the lower image-level may be attributed to our spatial anomaly detection at lower resolutions, which occasionally results in misclassification of slices due to imprecise tumor boundary detection. Nevertheless, when evaluating the volume as a whole rather than individual slices, our model effectively detects spatial anomalies, showcasing its robustness in comprehensive anomaly detection. The quantitative results for T1 volumes are also presented in the Table 4 for high-performance models, for further information.

**Table 3:** Quantitative results at the image level of all models on T2 volumes. This table presents the Quantitative results of all models at the image level, providing a comprehensive overview of each model’s effectiveness in anomaly detection. *Ours* denotes our method variant with Triplet Loss.

	AUROC	AUPRC	⌈ DICE⌉	Spec	ACC	Prec
SimpleNet	62.3	73.12	78.87	65.82	65.81	5.69
${\text{BGAD}}^{\text{w/o}}$	51.93	65.53	78.68	64.86	64.86	0
${\text{BGAD}}^{\text{100}}$	66.44	78.21	78.7	64.88	64.89	0.15
CFLOW-AD	51.02	64.35	78.03	63.98	63.98	0.09
EDC	**79.82**	**86.56**	**81.45**	**76.46**	**74.26**	**50.48**
DAE	67.85	77.87	78.86	66.92	66.62	12.42
AE-FLOW	63.74	73.12	78.72	65.3	65.26	2.84
CutPaste	55.77	71.04	78.73	64.92	64.92	0
PatchCore	71.1	78.82	79.1	68.03	67.79	19.26
RD++	74.57	81.2	79.94	70.34	70.02	29.01
3D_AE	69.91	79.47	79.44	68.43	68.22	19.42
Ours	75.31	84.57	79.5	69.05	68.67	22.48

**Table 4:** Quantitative results at the image level of all models on T1 volumes. This table presents the Quantitative results of all models at the image level, providing a comprehensive overview of each model’s effectiveness in anomaly detection. *Ours* denotes our method variant with Triplet Loss.

	AUROC	AUPRC	⌈ DICE⌉	Spec	ACC	Prec
${\text{BGAD}}^{\text{w/o}}$	61.79	48.90	63.51	47.68	55.93	29.45
EDC	69.98	55.17	64.70	49.67	59.16	36.41
PatchCore	72.06	54.50	66.15	51.67	62.05	41.86
RD++	84.19	71.85	74.54	67.27	76.96	**72.50**
3D_AE	83.80	69.72	75.71	64.95	76.53	66.92
Ours	**87.68**	**78.17**	**77.03**	**67.73**	**78.53**	71.26

### Localization qualitative results

**Fig 3:**
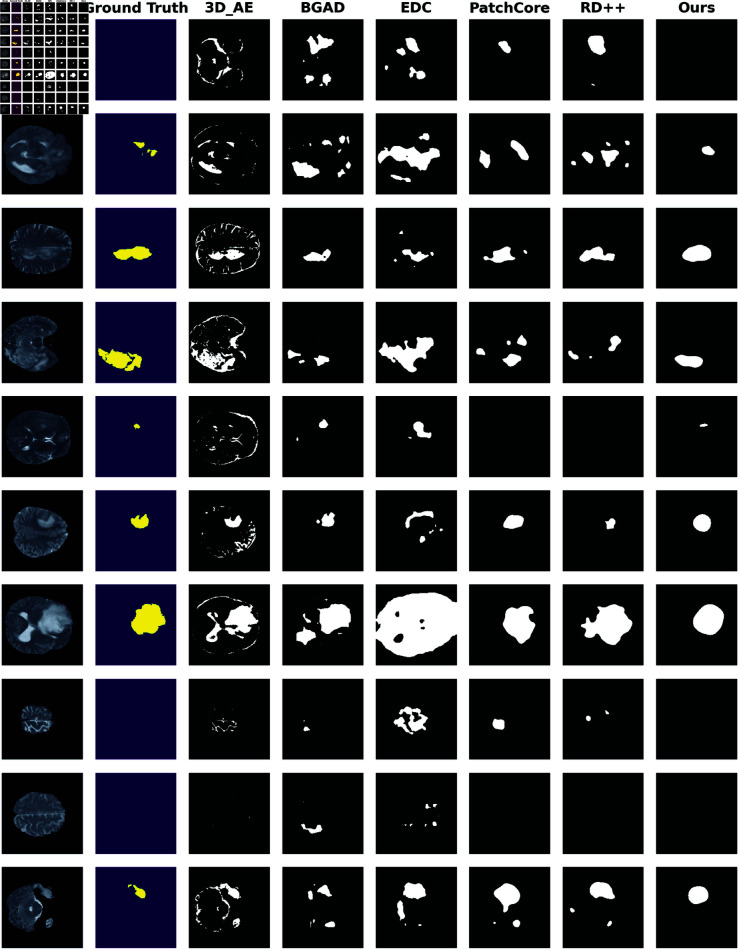
Qualitative Results on the SOTA methods on T2 volumes. This figure displays slices from volumes selected from the testing dataset, chosen to represent different levels within the volume and varied visual structures. This selection ensures a more comprehensive evaluation of the models’ performance. The first column presents the images (slices) from brain MRI volumes and the second column illustrates the ground-truth data for anomalous regions. The subsequent columns depict the binary anomaly map predicted by selected models. These maps are generated by applying the F1 threshold, which yield the best F1 score, to convert anomaly scores into binary masks.

Fig 3 presents the qualitative result for each model. The 2D and 3D reconstruction-based methods, **EDC** and **3D_AE** generate a significant number of false positives. Specifically, **EDC** often misses the central area of the anomaly, likely because this region is easier to reconstruct, whereas the transitional areas between normal and abnormal tissues pose more challenges to reconstruct. **BGAD**, while performing better, also generates some small false positives and the detected anomalies are not well-concentrated. **PatchCore** and **RD++** show improved performance but still produce some false positive regions. In contrast, our model accurately localizes that the anomalies without generating false positives away from the anomalous regions. Furthermore, the detected anomalous areas in our model encompass more true abnormal regions than those identified by other methods.

**Fig 4:**
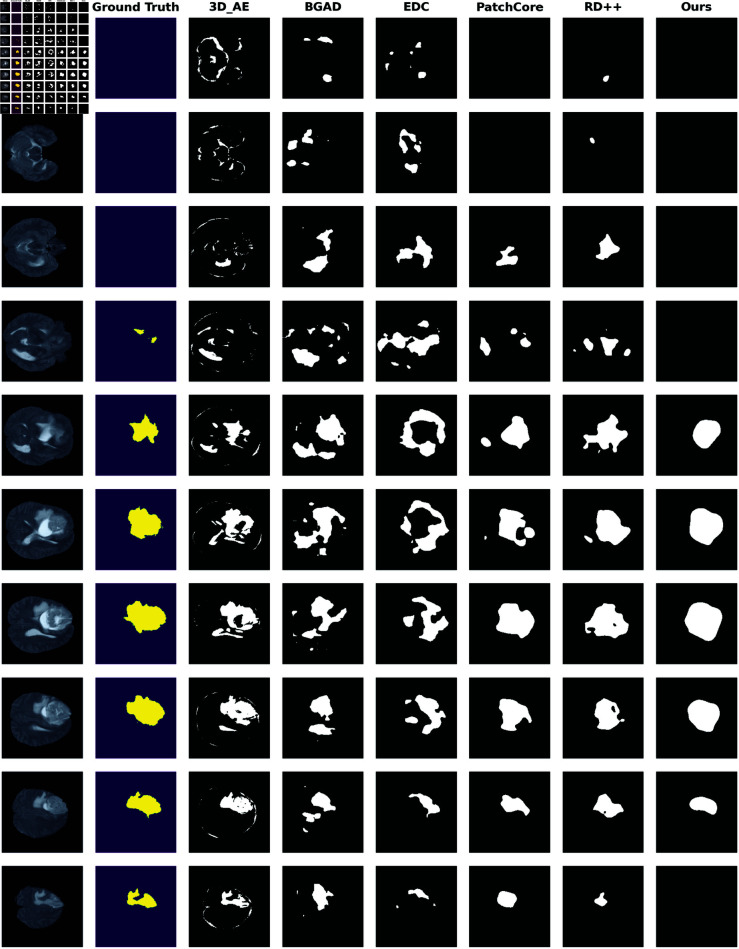
Qualitative Results on the SOTA methods on a single T2 volume. This figure displays intermediate slices in sequence from a single volume selected from the testing dataset. The visualization is designed to demonstrate how well the model performs spatially across an entire volume. The first row presents the images (slices) from brain MRI volumes and the second row shows the ground-truth for the anomalous regions. The remaining rows depict the binary anomaly maps predicted by SOTA models. These maps are generated by applying the F1 threshold, which achieves the best F1 score, to convert anomaly scores into binary masks.

Fig 4 displays the qualitative results with slices taken sequentially from a single volume. This visualization aids in assessing the spatial localization of anomalies. The other methods tend to generate small false positive regions, particularly in normal slices where anomalies slices where anomalies are erroneously detected. In contrast our method effectively localizes spatial anomalies without generating unnecessary false positives, leading to a more concise and straightforward predicted anomaly map. However, this focus on precise anomaly positioning and minimizing false positives compromises the ability to accurately delineate the anomaly’s boundaries. Consequently, the detected regions appear rough, occasionally missing some areas along the depth axis and misclassifying some slices. This limitation likely contributes to our method’s lower image-level metrics compared to those of **EDC**. We also plot the qualitative results of coronal slices in S1 Fig.

### Training process

**Fig 5:**
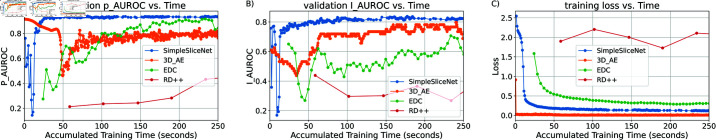
Plots of AUROC and loss during training. This figure presents plots of image-level and pixel-level AUROC, along with average training loss, observed during the training phase. For practicality, we trained on 60 volumes from the training dataset and used 20 volumes from the validation dataset for evaluation. We assessed model performance after processing each volume (every single iteration), and the results for the first 250 seconds are plotted. The interval between two consecutive points on a line indicates the time required for the model to process one volume, consisting of 96 slices. A: plot of pixel-level AUROC. B: plot of image-level AUROC. C: plot of average training loss.

Fig 5 illustrates that RD++ requires the longest time to process a brain MRI volume and to converge. It’s evident from the plot that our method converges more quickly and demonstrates greater stability compared to the other three methods evaluated.

### Computational cost

In Table 5, we compare those well-performing models in terms of memory size and inference time. This table shows that our methods has the smallest number of trainable parameters, which contributes to a shorter convergence time. Additionally, it requires less estimated memory and has a shorter inference time. While our inference time is slower than that of **EDC** and our estimated memory size is smaller than that of **PatchCore**, the differences are marginal. Moreover, **PatchCore** necessitates extensive additional memory to store the memory bank and a longer inference time to search for the nearest neighbors within the bank. **EDC**, while faster, shows inferior performance in localization tasks and has a larger number of trainable parameters.

**Table 5:** Estimate memory size and inference time. *Trainable* refers to the number of parameters in the model that can be updated during training, expressed in millions. *Non-trainable* denotes the number of parameters that remain fixed during training, also in millions. *Memory Size* indicates the estimated amount of memory required to store both trainable and non-trainable parameters, measured in megabytes. *inference time* describes the time needed for the model to compute the anomaly map for a volume comprising 96 slices, with the time measured in seconds.

	Trainable	Non-trainable	Memory Size	Inference time
EDC	137m	0	525.58mb	0.34s
PatchCore	0	68.88m	262.77mb	1.94s
RD++	107.69m	68.88m	673.5mb	2.18s
3D_AE	235.82m	0	899.57mb	1.25s
Ours	3.2m	69.93m	269.41mb	0.74s

### Ablation study

#### Study on contrastive losses

To investigate the impact of different contrastive losses on softly refining the boundary of normal features, we evaluate three candidate losses: Pairwise Ranking Loss (PRL) [31], Triplet Loss (TL) [30], and Boundary-guided Semi-Push-Pull Loss (BG-SPP) [7]. These losses share a common objective: pulling normal features closer while softly pushing anomalous features away with a margin. Following Loss function, given log probabilities of normal features $\log p_{i}$ and abnormal features $\log q_{j}$, the BG-SPP loss is defined as:

$${ℒ}_{bg-spp}=\sum_{i=1}^{N}\left|min((\log p_{i}-b_{n}),0)\right|+\sum_{j=1}^{M}\left|max((\log q_{j}-b_{n}+\tau ),0)\right|,$$ (12)

where *b*_*n*_ is the normal boundary obtained by selecting the β-th percentile of the sorted normal log-likelihood distribution, the τ is the margin between the boundaries of the normal and abnormal features.

The PRL loss is formulated as:

$$\underset{pair-ranking}{ℒ}=\sum_{i,j}max(0,\tau -(\log p_{i}-\log q_{j}))$$ (13)

Table 6 illustrates the detection and localization performance associated with different contrastive loss configurations. The comparison between the first three rows and the last row in this table indicates that incorporating an additional contrastive loss significantly enhances performance. Among these three candidate losses, all show comparable performance, with the TL variant exhibiting a slight improvement.

**Table 6:** Quantitative results on different contrastive loss. *w/o CL* refers to our model variant without the integration of any additional CL loss. This configuration allows us to evaluate the impact of excluding the contrastive loss on the models performance.

	Image-level			Pixel-level			
	AUROC	AUPRC	⌈ DICE⌉	AUROC	AUPRC	PRO	⌈ DICE⌉
w/ TL	75.31	84.57	79.5	95.55	51.33	76.98	51.07
w/ PRL	74.08	83.95	79.29	94.94	47.35	75.13	47.87
w/ BG-SPP	73.8	82.67	79.67	95.8	53.32	75.83	52.8
w/o CL	69.27	80.34	79.15	92.43	34.8	67.6	38.46

#### Study on components

In Table 7, we investigate the significance of each component to address questions such as: Does aggregation on axial slices diminish information? Does processing axial slices independently in the feature extractor limit the expressiveness of the resulting 3D feature volume? Can a pre-trained model tailored to brain MRI datasets enhance performance? Why is NF more effective than hard classification by a simple binary classification model? The table demonstrates that each modification significantly improves performance. Specifically, the comparison between the first and last row indicates that aggregation effectively utilizes axial information to enhance the model. However, it remains unclear whether the aggregated features are representative or contain irrelevant information, a common issue in embedding-based methods where feature extraction and anomaly detection are distinct processes. The comparison between the second and last row suggests that using a pre-trained model more attuned to brain MRI dataset is beneficial. For a fair comparison, we fine-tuned a pre-trained ImageNet model on an additional dataset of low quality and quantity to limit extraneous knowledge. We explored various methods to embed the extracted features into a space suitable for brain MRI dataset, including fine-tuning during training and adding a projection layer as done by SimpeNet [6]. Our modified pre-finetuned feature extractor with **EDC** achieved the best performance, stable training, and reduced training time. Additionally, we compare our model to a special case of SimpleNet with aggregation. This comparison further highlights the effectiveness of the CNF with SPP mechanism.

**Table 7:** Quantitative results of our method with different modifications. This table illustrates the performance of our model with the removal of each component, assessed using both pixel- and image-level metrics. *Ours w/o Agg.* refers to our model without the part the slices’ feature aggregating component, essentially transforming it into a 2D version that processes brain MRI data slice by slice. *Ours w/o EDC* indicates that the feature extractor was not fine-tuned, with the extracted features directly applied to anomaly detection tasks. *SimpleNet w/ Agg.* represents a modification where our CNF is replaced with a binary classification model, effectively making it a 3D version of SimpleNet that utilizes our aggregation approach. Finally, *Ours* denotes our proposed method with Triplet Loss.

	Image-level			Pixel-level			
	AUROC	AUPRC	⌈ DICE⌉	AUROC	AUPRC	PRO	⌈ DICE⌉
Ours w/o Agg.	70.81	80.27	79.04	94.58	43.99	68.51	46.14
Ours w/o EDC	65.03	75.19	78.93	93.67	37.88	72.54	41.1
SimpleNet w/ Agg.	60.13	72.78	78.82	86.24	21.63	57.82	25.96
Ours	75.31	84.57	79.5	95.55	51.33	76.98	51.07

## Discussion

In this study, we introduced SimpleSliceNet, an efficient framework designed to process both 2D and 3D brain MRI data for anomaly detection and localization. SimpleSliceNet leverages a backbone network to extract normal features and introduces Gaussian noise to these features to simulate anomalies. It utilizes a conditional NF combined with Triplet Loss to effectively delineate the boundaries of the normal distribution, adapting to the unique characteristics of brain MRI data. The quantitative detection and localization analyses presented in this paper offer valuable insights into the efficacy of different anomaly detection models in medical imaging, particularly in brain MRI. These findings highlight the strengths and limitations of our proposed model compared to other SOTA methods.

Unsupervised methods for images fall into three categories: reconstruction-based, synthesizing-based, and embedding-based. Reconstruction-based models are commonly used in brain MRI anomaly detection, while synthesizing-based and embedding-based approaches are less explored in this field.

**Reconstruction-based** methods, including auto-encoder (AE) [24,34] and Generative Adversarial Network (GAN) [35], are extensively studied and considered SOTA in medical imaging. These models are trained solely on normal data, assuming anomalies cannot be accurately reconstructed, making the residual difference between input and reconstructed samples indicative of anomalies. For instance, in [10], the authors fine tuned a pre-trained network as an encoder and trained a decoder with a reversed architecture to reconstruct medical images, using contrastive loss to maintain the discriminative ability. Similarly, [36] used an AE framework to identify chronic brain infarcts on MRI, employing an additional decoder and discriminator for anomaly detection at the image level instead of directly taking the maximum anomaly scores on pixels. Some approaches, such as [32,37], utilize NF and diffusion models to compute anomaly likelihood on latent representations instead of pixel-level scores. Despite their utility, reconstruction-based models often produces blurry and low-quality images, and there is a trade-off between reconstruction ability and model generality, necessitating careful experimentation to balance these factors. Additionally, these methods generate noisy anomaly maps that require further post-processing steps. For medical data, the variation among normal samples (e.g. due to differences between patients or imaging infrastructures) may be greater than the variation of anomalous samples. In such cases, reconstruction methods that focus on deviations may not be effective and may create too many false positives. Our method aims to reduce false positives while maintaining performance. Qualitatively, while **EDC** and **3D_AE** tend to generate a high number of positives and struggle with anomaly localization, our model consistently demonstrates superior accuracy in detecting true anomalies with minimal false positives. This precision is critical in clinical settings, correctly identifying anomalous regions can significantly impact diagnosis and treatment plans. However, the focus on minimizing false positives introduces challenges in precisely delineating the anomaly’s boundaries, occasionally resulting in rough detected regions and misclassification of some slices.

**Synthesizing-based** methods offer an alternative approach to anomaly detection by generating realistic anomalies from normal images. In [33], small patches are cut from other normal images and pasted onto normal samples. To ensure smooth boundaries, NSA [38] integrated Poisson image editing to create smooth and continuous boundaries on synthesized anomalies. However, synthesized anomalies may not accurately represent real anomalies due to the unpredictable nature of anomalies within dataset, particular in tasks like brain MRI anomaly detection. We followed SimpleNet [6] to generate deviations within features to create anomalies, which may be more suitable for capturing a wider range of anomalies.

**Embedding-based** methods involve learning the normal features of data and projecting them into an embedding space where abnormal features are further from normal ones. Many STOA models currently use pre-trained ImageNet models to extract meaningful features and save training time. SimpleNet [6] utilizes an ImageNet-pretrained encoder to extract multi-scale features, synthesize anomalies by adding Gaussian noise to the normal features, and trains a simple discriminator to classify the features. NF [29] is used to convert the extracted features into a desired distribution, typically a Gaussian distribution. CFLOW-AD [2] employs an ImageNet-pretrained encoder to extract features from intermediate layers and uses conditional NF (CNF) for each layer to obtain the normal distribution, enabling the detection of anomalous features at different scales. BGAD [7] aims to establish a clearer boundary on the normal distribution with a small number of anomalous examples through a soft pushing mechanism. These methods have achieved SOTA performance compared to other types of approach. Inspired by SimpleNet and BGAD, our method is specifically designed to address the challenges of medical data by generating synthetic anomalies at the feature level. This approach helps refine the normal boundary without disrupting the diverse normal distribution present in medical data.

**Knowledge Distillation** methods [1,9,11,12] also utilize pre-trained models, training a Student network to learn the knowledge specific to normal data from a Teacher network, ensuring that the Student network only possesses knowledge about normal features. The residual between the output from Student and Teacher reveals anomaly information. However, these methods often demand significant memory and entail long training times. Additionally, for brain MRI data, there is a lack of suitable pre-trained models. To mitigate training workload and time, we still prefer models pre-trained on ImageNet to extract features, and investigate a better way to leverage pre-trained models on medical data.

For 3D brain MRI volumes, reconstruction-based models represent the SOTA unsupervised approach for detecting anomalies. [13,14] used the idea of reconstruction to identify anomalies in these volumes. Instead of relying on the use of maximum pixel scores for detection in [13,14] used autoregressive transformers to analyze the distribution of the latent vector. Meanwhile, [15,16] are supervised methods adopted 3D U-Net [39] and 3D ResU-Net [40] frameworks respectively to integrate feature maps from different resolution levels. However, these 3D CNN networks demand significant memory for parameter storage and considerable time for backpropagation. Instead, our model showcases computational efficiency. It requires fewer trainable parameters, operates with less memory, and achieves faster convergence. This efficiency does not compromise performance, which is pivotal for deploying these models in real-world clinical settings where processing speed and resource utilization are crucial.

Our model excels in several critical metrics such as AUROC, PRO, and ⌈DICE⌉ metrics, which are essential for understanding the effectiveness of anomaly detection in imbalanced datasets. Despite the competitive performance of models like **PatchCore** and **RD++** on AUROC, our method’s advancements in these specific metrics underscore its robustness and suitability for clinical applications that require precise anomaly delineation. Conversely, despite **EDC** showing outstanding performance across standard image-level metrics, its shortcomings in localization and stability - evidenced by both quantitative and qualitative evaluations - point to limitations in its practical applicability for consistent anomaly detection. Our model’s superior tumor detection capability, though associated with lower image-level scores than **EDC**, can be attributed to our approach to spatial anomaly detection. While this approach reduces resolution to manage computational demands, it may lead to occasional misclassifications, especially at the boundaries of anomalous regions. However, they are significantly mitigated when considering the volumetric assessment of anomalies, where our model excel in detecting spatial discrepancies across the entire volume. These findings emphasize the need for future research to focus on enhancing resolution and boundary clarity in anomaly detection models without compromising on the computational efficiency required for real-time applications.

The component analysis reveals which aspects of the model architecture contribute most to its success and identifies areas needing refinement. A remaining question is whether the aggregated features representative for anomaly detection and if there are any other ways to better utilize depth-wise information. We conducted a preliminary experiment with depth-wise attention module, which showed promise result but required additional training time and computation resources. This area warrants further investigation. Our results also demonstrate the importance of a pre-trained feature extractor that is well-adapted to our target space. A pre-trained model with robust feature learning capabilities in the medical or even brain MRI domain can significantly enhance disease monitoring in the medical field.

Future work could explore integrating advanced deep learning architectures that improve resolution handling without substantially increasing computational requirements. Further investigations into the training processes might also yield improvements in model stability, particularly for methods like **EDC** that show potential but are hindered by specific deficiencies. Looking forward, several avenues for enhancing SimpleSliceNet could be explored. Future research could investigate integrating a more sophisticated pre-trained network better tailored to the nuances of medical imaging data. Additionally, advancing fine-tuning techniques and utilizing higher quality datasets could further refine the model’s performance. Another promising area of development is improving the method of aggregating depth-wise information, which could lead to even more precise and effective anomaly detection.

Our evaluations demonstrate that SimpleSliceNet surpasses several SOTA methods in detecting anomalies and outperforms traditional 3D reconstruction-based methods in 3D brain MRI volumes. Notably, it achieves this superior performance with significantly reduced computational time and memory usage. These results highlight SimpleSliceNet’s potential as a robust tool for clinical applications, where efficiency and accuracy are paramount.

## Human subjects data

In this work, we used three publicly available medical datasets including BraTS2021, Br35H, and IXI. These datasets do not contain any personally identifiable information about patients, ensuring compliance with privacy standards. We have cited these datasets in the reference section to acknowledge their source and usage appropriately.Supporting information

## Supporting information

S1 Fig Anomaly localization across consecutive coronal slices of a 3D T2 volume. See Fig 4 for description of this figure.
(TIFF)

S2 Fig Anomaly localization on coronal slices across all T1 volumes. See Fig 3 for description of this figure.
(TIFF)

S3 Fig Anomaly localization across consecutive axial slices of a 3D T1 volume. See Fig 4 for description of this figure.
(TIFF)

S4 Fig Anomaly localization across consecutive coronal slices of a 3D T1 volume. See Fig 4 for description of this figure.
(TIFF)

## References

[1] Zhang X, Li S, Li X, Huang P, Shan J, Chen T. DeSTSeg: segmentation guided denoising student-teacher for anomaly detection. In: Proceedings of the IEEE/CVF Conference on Computer Vision and Pattern Recognition. 2023, pp. 3914–23.

[2] Gudovskiy D, Ishizaka S, Kozuka K. Cflow-ad: Real-time unsupervised anomaly detection with localization via conditional normalizing flows. In: Proceedings of the IEEE/CVF Winter Conference on Applications of Computer Vision. 2022, pp. 98–107.

[3] Guo H, Ren L, Fu J, Wang Y, Zhang Z, Lan C, et al. Template-guided hierarchical feature restoration for anomaly detection. In: Proceedings of the IEEE/CVF International Conference on Computer Vision. 2023, pp. 6447–58.

[4] Jewell JT, Khazaie VR, Mohsenzadeh Y. One-class learned encoder-decoder network with adversarial context masking for novelty detection. In: Proceedings of the IEEE/CVF Winter Conference on Applications of Computer Vision. 2022, pp. 3591–601.

[5] Bergmann P, Fauser M, Sattlegger D, Steger C. MVTec AD—a comprehensive real-world dataset for unsupervised anomaly detection. In: Proceedings of the IEEE/CVF conference on computer vision and pattern recognition. 2019, pp. 9592–600.

[6] Liu Z, Zhou Y, Xu Y, Wang Z. Simplenet: A simple network for image anomaly detection and localization. In: Proceedings of the IEEE/CVF Conference on Computer Vision and Pattern Recognition. 2023, pp. 20402–11.

[7] Yao X, Li R, Zhang J, Sun J, Zhang C. Explicit boundary guided semi-push-pull contrastive learning for supervised anomaly detection. In: Proceedings of the IEEE/CVF Conference on Computer Vision and Pattern Recognition. 2023, pp. 24490–9.

[8] Roth K, Pemula L, Zepeda J, Schölkopf B, Brox T, Gehler P. Towards total recall in industrial anomaly detection. In: Proceedings of the IEEE/CVF Conference on Computer Vision and Pattern Recognition. 2022, pp. 14318–28.

[9] Salehi M, Sadjadi N, Baselizadeh S, Rohban MH, Rabiee HR. Multiresolution knowledge distillation for anomaly detection. In: Proceedings of the IEEE/CVF Conference on Computer Vision and Pattern Recognition. 2021, pp. 14902–12.

[10] Guo J, Lu S, Jia L, Zhang W, Li H. Encoder-decoder contrast for unsupervised anomaly detection in medical images. IEEE Trans Med Imaging. 2023;43(3):1102–12. 10.1109/TMI.2023.3327720 37883280

[11] Deng H, Li X. Anomaly detection via reverse distillation from one-class embedding. In: Proceedings of the IEEE/CVF Conference on Computer Vision and Pattern Recognition. 2022, pp. 9737–46.

[12] Tien TD, Nguyen AT, Tran NH, Huy TD, Duong S, Nguyen CDT, et al. Revisiting reverse distillation for anomaly detection. In: Proceedings of the IEEE/CVF Conference on Computer Vision and Pattern Recognition. 2023, pp. 24511–20.

[13] Luo G, Xie W, Gao R, Zheng T, Chen L, Sun H. Unsupervised anomaly detection in brain MRI: learning abstract distribution from massive healthy brains. Comput Biol Med. 2023;154:106610. doi: 10.1016/j.compbiomed.2023.106610 36708653

[14] Pinaya WHL, Tudosiu P-D, Gray R, Rees G, Nachev P, Ourselin S, et al. Unsupervised brain imaging 3D anomaly detection and segmentation with transformers. Med Image Anal. 2022;79:102475. doi: 10.1016/j.media.2022.102475 35598520 PMC10108352

[15] Rudie JD, Weiss DA, Colby JB, Rauschecker AM, Laguna B, Braunstein S, et al. Three-dimensional U-net convolutional neural network for detection and segmentation of intracranial metastases. Radiol Artif Intell. 2021;3(3):e200204. doi: 10.1148/ryai.2021200204 34136817 PMC8204134

[16] Li H, Chen M, Wang J, Illapani VSP, Parikh NA, He L. Automatic segmentation of diffuse white matter abnormality on T2-weighted brain MR images using deep learning in very preterm infants. Radiol Artif Intell. 2021;3(3):e200166. doi: 10.1148/ryai.2021200166 34142089 PMC8166113

[17] Gupta U, Lam PK, Ver Steeg G, Thompson PM. Improved brain age estimation with slice-based set networks. In: 2021 IEEE 18th International Symposium on Biomedical Imaging (ISBI). 2021, pp. 840–4.

[18] Gupta U, Chattopadhyay T, Dhinagar N, Thompson PM, Ver Steeg G. Transferring models trained on natural images to 3D MRI via position encoded slice models. In: 2023 IEEE 20th International Symposium on Biomedical Imaging (ISBI). IEEE; 2023, pp. 1–5.

[19] Hamada A. Br35H: brain tumor detection. Available from: https://www.kaggle.com/datasets/ahmedhamada0/brain-tumor-detection. 2020.

[20] IXI Dataset. Available from: https://brain-development.org/ixi-dataset/.

[21] Baid U, Ghodasara S, Mohan S, Bilello M, Calabrese E, Colak E, et al. The rsna-asnr-miccai brats 2021 benchmark on brain tumor segmentation and radiogenomic classification. arXiv, preprint, 2021. arXiv:210702314. https://arxiv.org/abs/2107.02314

[22] Bakas S, Akbari H, Sotiras A, Bilello M, Rozycki M, Kirby JS, et al. Advancing the cancer genome atlas glioma MRI collections with expert segmentation labels and radiomic features. Sci Data. 2017;4:170117. doi: 10.1038/sdata.2017.117 28872634 PMC5685212

[23] Menze BH, Jakab A, Bauer S, Kalpathy-Cramer J, Farahani K, Kirby J, et al. The multimodal Brain Tumor Image Segmentation Benchmark (BRATS). IEEE Trans Med Imaging. 2015;34(10):1993–2024. doi: 10.1109/TMI.2014.2377694 25494501 PMC4833122

[24] Kascenas A, Sanchez P, Schrempf P, Wang C, Clackett W, Mikhael SS, et al. The role of noise in denoising models for anomaly detection in medical images. Med Image Anal. 2023;90:102963. doi: 10.1016/j.media.2023.102963 37769551

[25] Isensee F, Schell M, Pflueger I, Brugnara G, Bonekamp D, Neuberger U, et al. Automated brain extraction of multisequence MRI using artificial neural networks. Hum Brain Mapp. 2019;40(17):4952–64. doi: 10.1002/hbm.24750 31403237 PMC6865732

[26] Jenkinson M, Smith S. A global optimisation method for robust affine registration of brain images. Med Image Anal. 2001;5(2):143–56. doi: 10.1016/s1361-8415(01)00036-6 11516708

[27] Jenkinson M, Bannister P, Brady M, Smith S. Improved optimization for the robust and accurate linear registration and motion correction of brain images. Neuroimage. 2002;17(2):825–41. doi: 10.1016/s1053-8119(02)91132-8 12377157

[28] Deng J, Dong W, Socher R, Li LJ, Li K, Fei-Fei L. Imagenet: a large-scale hierarchical image database. In: 2009 IEEE Conference on Computer Vision and Pattern Recognition. 2009, pp. 248–55.

[29] Dinh L, Sohl-Dickstein J, Bengio S. Density estimation using real nvp. arXiv, preprint, 2016. https://arxiv.org/abs/1605.08803

[30] Schroff F, Kalenichenko D, Philbin J. Facenet: A unified embedding for face recognition and clustering. In: Proceedings of the IEEE Conference on Computer Vision and Pattern Recognition. 2015, pp. 815–23.

[31] Chopra S, Hadsell R, LeCun Y. Learning a similarity metric discriminatively, with application to face verification. In: 2005 IEEE Computer Society Conference on Computer Vision and Pattern Recognition (CVPR’05), vol. 1. IEEE; 2005, pp. 539–46. doi: 10.1109/cvpr.2005.202

[32] Zhao Y, Ding Q, Zhang X. AE-FLOW: autoencoders with normalizing flows for medical images anomaly detection. In:The Eleventh International Conference on Learning Representations. 2022.

[33] Li CL, Sohn K, Yoon J, Pfister T. Cutpaste: Self-supervised learning for anomaly detection and localization. In: Proceedings of the IEEE/CVF Conference on Computer Vision and Pattern Recognition. 2021, pp. 9664–74.

[34] Kascenas A, Pugeault N, O’Neil AQ. Denoising autoencoders for unsupervised anomaly detection in brain MRI. In: International Conference on Medical Imaging with Deep Learning. PMLR; 2022, pp. 653–64.

[35] Schlegl T, Seeböck P, Waldstein SM, Langs G, Schmidt-Erfurth U. f-AnoGAN: fast unsupervised anomaly detection with generative adversarial networks. Med Image Anal. 2019;54:30–44. doi: 10.1016/j.media.2019.01.010 30831356

[36] van Hespen KM, Zwanenburg JJM, Dankbaar JW, Geerlings MI, Hendrikse J, Kuijf HJ. An anomaly detection approach to identify chronic brain infarcts on MRI. Sci Rep. 2021;11(1):7714. doi: 10.1038/s41598-021-87013-4 33833297 PMC8032662

[37] Pinaya WH, Graham MS, Gray R, Da Costa PF, Tudosiu PD, Wright P, et al. Fast unsupervised brain anomaly detection and segmentation with diffusion models. In: International Conference on Medical Image Computing and Computer-Assisted Intervention. Springer; 2022, pp. 705–14.

[38] Schlüter HM, Tan J, Hou B, Kainz B. Natural synthetic anomalies for self-supervised anomaly detection and localization. In: European Conference on Computer Vision. Springer; 2022, pp. 474–89.

[39] Çiçek Ö, Abdulkadir A, Lienkamp SS, Brox T, Ronneberger O. 3D U-Net: learning dense volumetric segmentation from sparse annotation. In: Medical Image Computing and Computer-Assisted Intervention–MICCAI 2016: 19th International Conference, Athens, Greece, October 17–21, 2016, Proceedings, Part II 19. Springer; 2016, pp. 424–32.

[40] Lee K, Zung J, Li P, Jain V, Seung HS. Superhuman accuracy on the SNEMI3D connectomics challenge. arXiv, preprint, 2017. doi: 10.48550/arXiv.1706.00120

