# Supplementary figures and images for: Efficient slice anomaly detection network for 3D brain MRI Volume
Source: PLOS Digit Health. 2025 Jun 20;4(6):e0000874. doi: 10.1371/journal.pdig.0000874 (PMC12180662; doi:10.1371/journal.pdig.0000874)

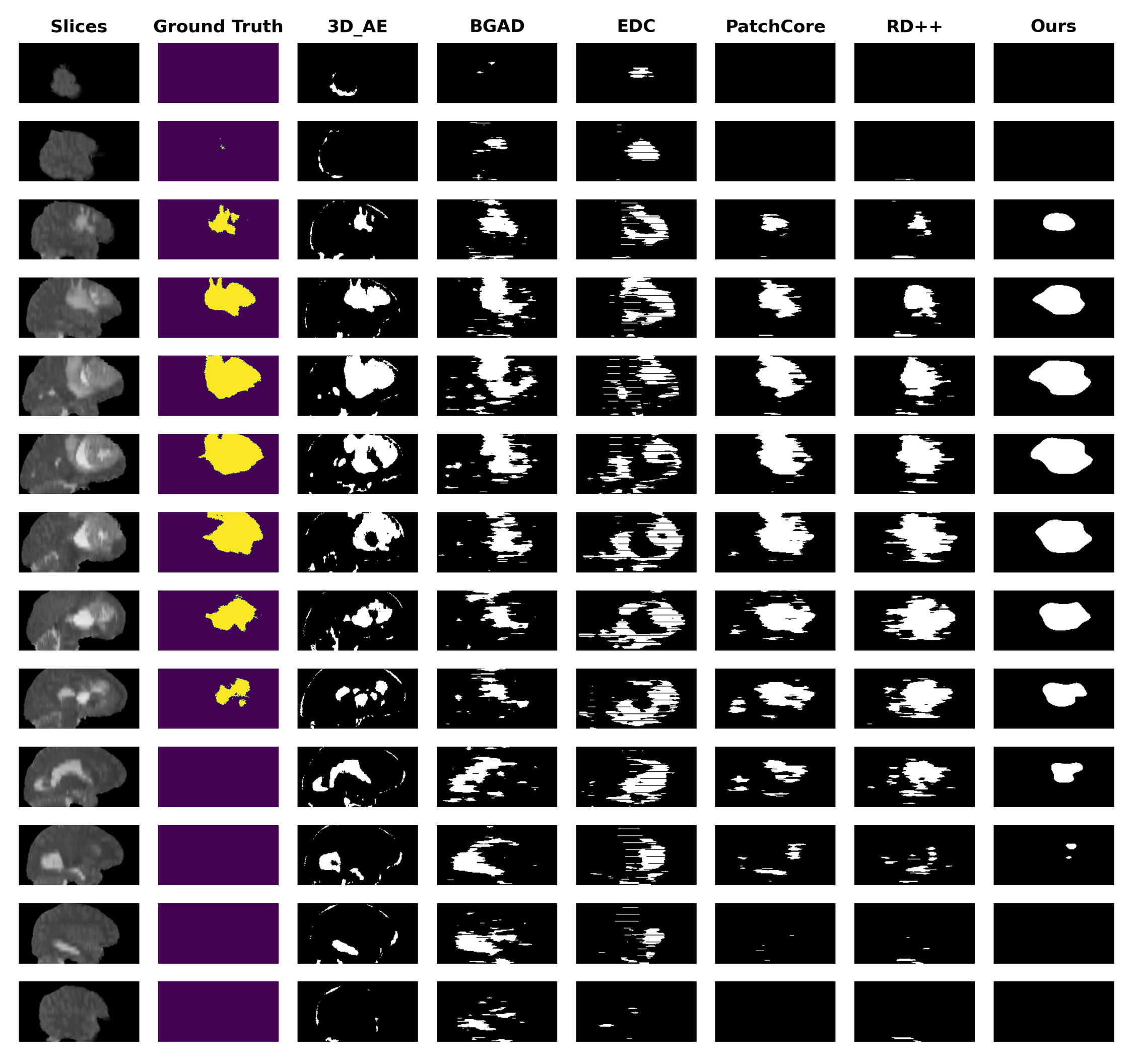

Supplement: S1 Fig — See Fig 4 for description of this figure. (TIFF) [file pdig.0000874.s001.tif]

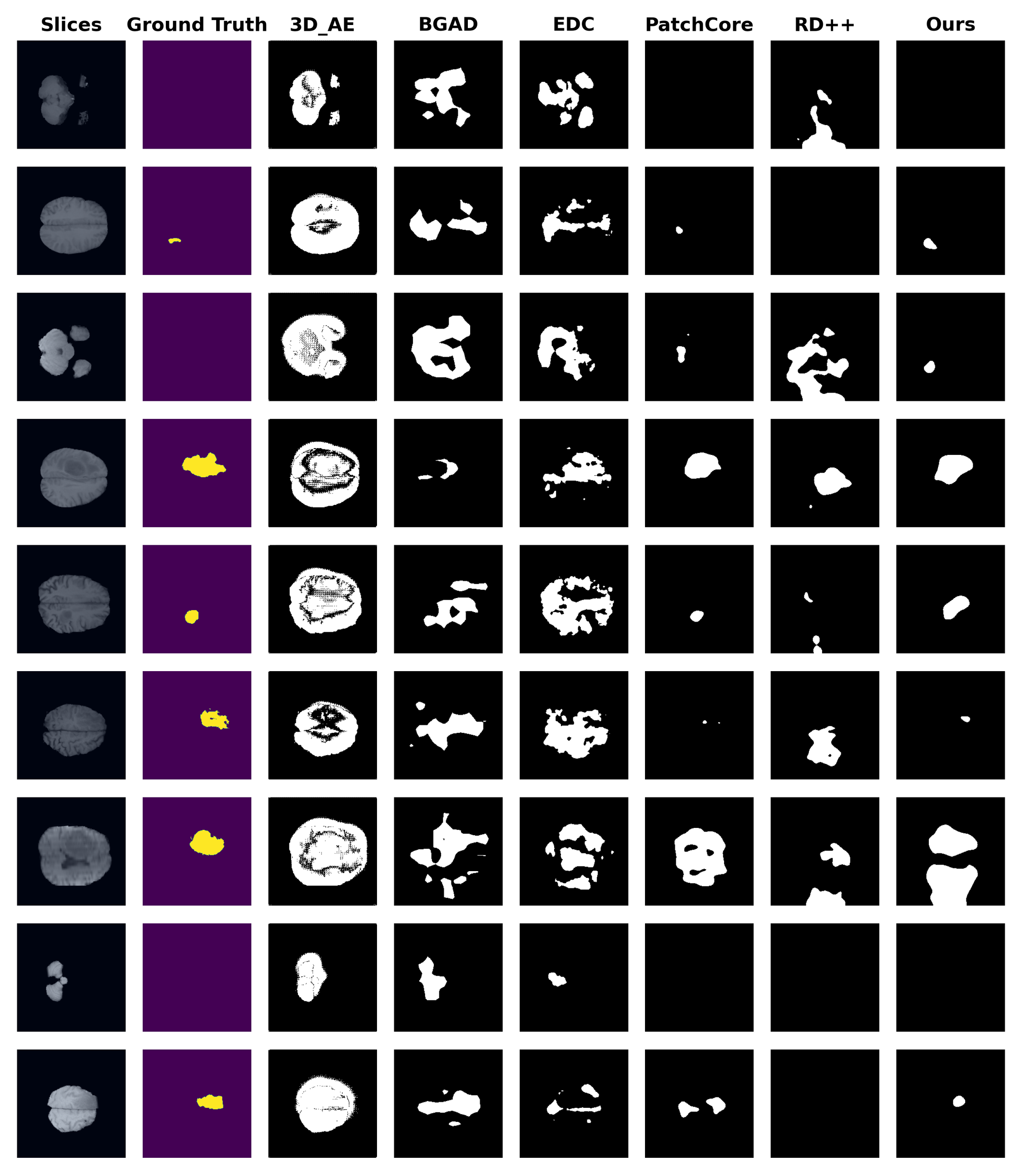

Supplement: S2 Fig — See Fig 3 for description of this figure. (TIFF) [file pdig.0000874.s002.tif]

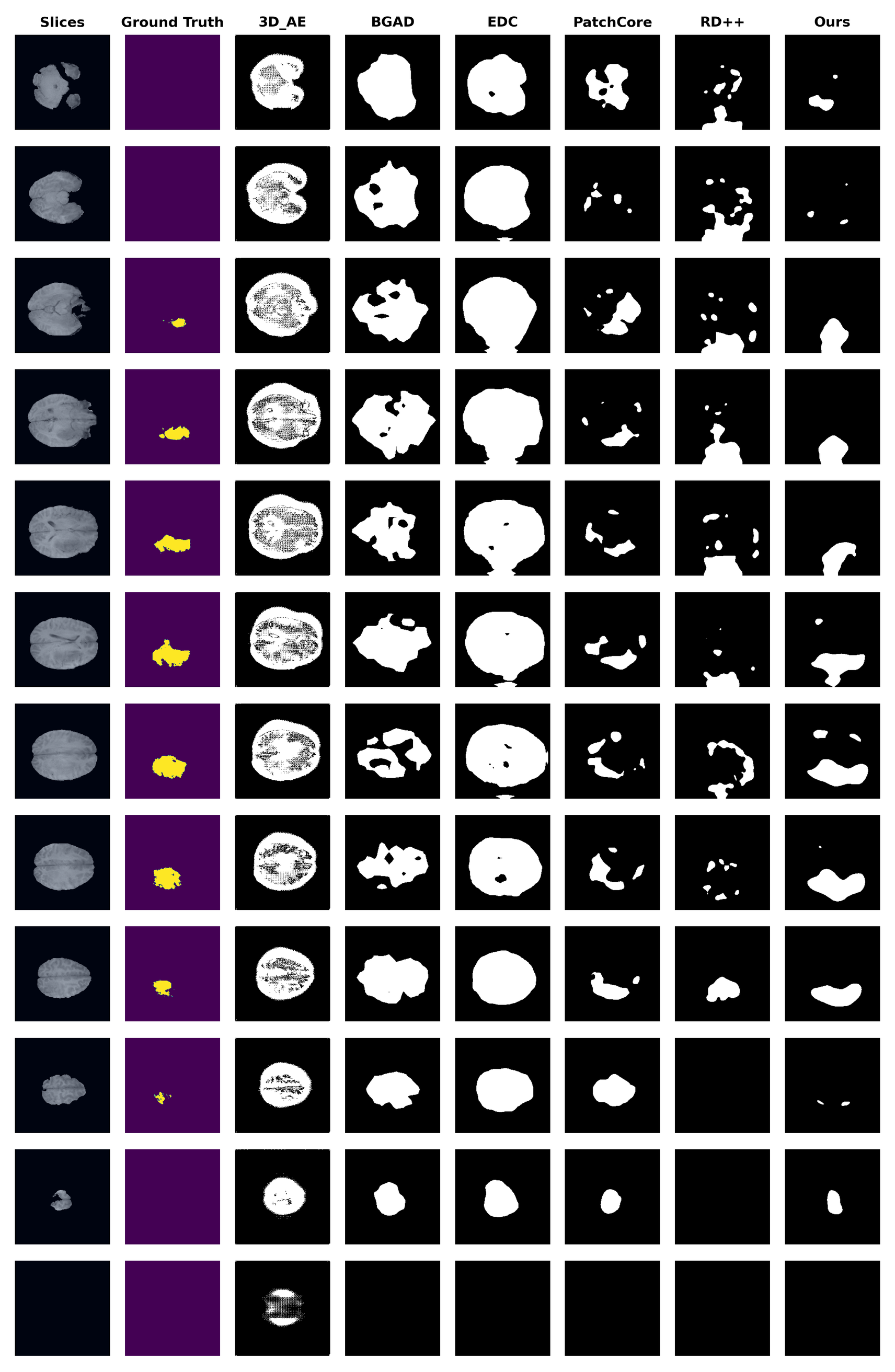

Supplement: S3 Fig — See Fig 4 for description of this figure. (TIFF) [file pdig.0000874.s003.tif]

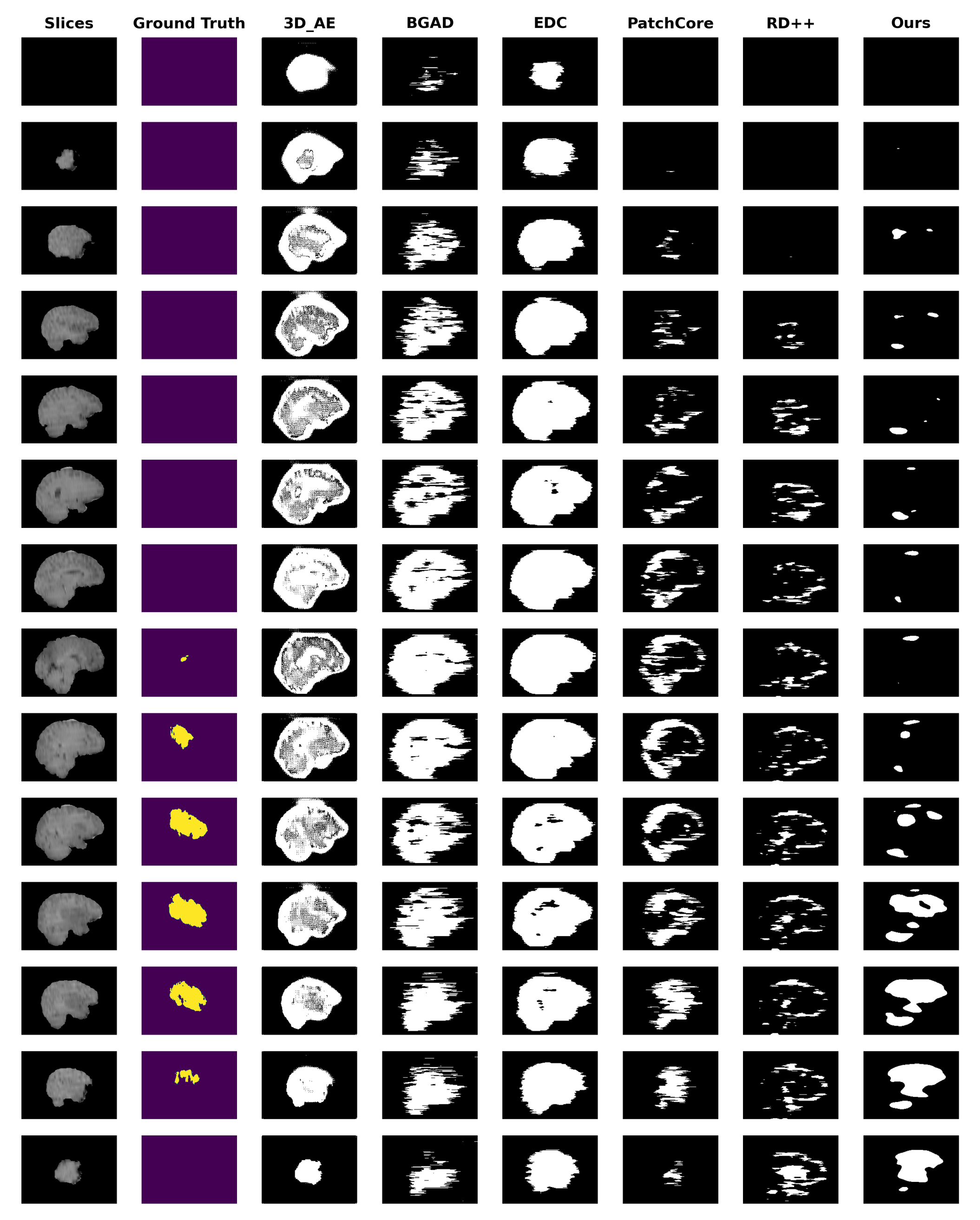

Supplement: S4 Fig — See Fig 4 for description of this figure. (TIFF) [file pdig.0000874.s004.tif]
